# Δ9-Tetrahydrocannabivarin (THCV): a commentary on potential therapeutic benefit for the management of obesity and diabetes

**DOI:** 10.1186/s42238-020-0016-7

**Published:** 2020-01-31

**Authors:** Amos Abioye, Oladapo Ayodele, Aleksandra Marinkovic, Risha Patidar, Adeola Akinwekomi, Adekunle Sanyaolu

**Affiliations:** 1 Lloyd L. Gregory School of Pharmacy, Palm Beach Atlantic University, West Palm Beach, Florida USA; 2 Saint James School of Medicine, The Quarter, Anguilla; 3 Federal Ministry of Health, Abuja, Nigeria

**Keywords:** Δ9-Tetrahydrocannabivarin (THCV), Tetrahydrocannabinol (THC), *Cannabis sativa* (marijuana), Obesity, Diabetes

## Abstract

Δ9-Tetrahydrocannabivarin (THCV) is a cannabis-derived compound with unique properties that set it apart from the more common cannabinoids, such as Δ9-tetrahydrocannabinol (THC). The main advantage of THCV over THC is the lack of psychoactive effects. In rodent studies, THCV decreases appetite, increases satiety, and up-regulates energy metabolism, making it a clinically useful remedy for weight loss and management of obesity and type 2 diabetic patients. The distinctions between THCV and THC in terms of glycemic control, glucose metabolism, and energy regulation have been demonstrated in previous studies. Also, the effect of THCV on dyslipidemia and glycemic control in type 2 diabetics showed reduced fasting plasma glucose concentration when compared to a placebo group. In contrast, THC is indicated in individuals with cachexia. However, the uniquely diverse properties of THCV provide neuroprotection, appetite suppression, glycemic control, and reduced side effects, etc.; therefore, making it a potential priority candidate for the development of clinically useful therapies in the future. Hopefully, THCV could provide an optional platform for the treatment of life-threatening diseases.

## Background

The therapeutic benefits of the extracts from the plant *Cannabis sativa L.* and its subspecies (hemp, marijuana) have been extensively studied. Cannabidiol (CBD), Δ-9-tetrahydrocannabinol (THC) and Δ-9-tetrahydrocannabivarin (THCV) are the major components isolated from *Cannabis sativa* and have been reported extensively in modern literature. THC is the primary psychoactive component of *Cannabis sativa* and its medicinal properties are attributed to its specific interaction with the endocannabinoid system (ECS) (Borgelt et al. 2013; McPartland et al. 2015; Chakrabarti et al. 2015). ECS consists of two types of endogenous G protein-coupled cannabinoid receptors (CB_1_ and CB_2_) that are located in the mammalian brain and throughout the central and peripheral nervous systems (Pertwee 2008; Solinas et al. 2008). The EC system represents a major neuromodulatory system involved in the regulation of emotional responses, behavioral reactivity, and social interactions. Pathophysiologic manipulation of the ECS has been exploited as a key tool in the management of severe disease conditions of the central nervous system. For example, in recent years, elements of the ECS and its pathways have been explored as therapeutic measures for mitigating some central nervous system diseases such as Autism Spectrum Disorder (ASD) and epilepsy (Chakrabarti et al. 2015). The endocannabinoid system is also responsible for the maintenance of energy homeostasis and the regulation of lipid and glucose metabolism (McPartland et al. 2015). In the same vein, molecular markers have been identified in the ECS membrane transporters (AM404) that could trigger autistic behavior when the cannabinoid receptors are activated (Chakrabarti et al. 2015).

THC produces various psychoactive effects by activation of the CB_1_ cannabinoid receptors in the brain, especially the basal ganglia, substantia nigra, globus pallidus, hippocampus, cerebellum, etc. These locations indicate that THC is involved in the modulation of memory, emotions, and movement. Activation of the CB_1_ receptors leads to inhibition of adenylyl cyclase and blockade of voltage-operated calcium channels, which in turn suppresses neuronal excitability and inhibition of neurotransmission of serotonin (Pertwee 2008). Therefore, the therapeutic benefits of THC include the management of conditions associated with depression, Parkinson’s disease, Alzheimer’s disease, resistant childhood seizures, chronic pain, multiple sclerosis, convulsions, glaucoma, neuropathic pain and a variety of other conditions (Hill 2015; Grant et al. 2012). It is important to note that *Cannabis sativa* is not a miracle plant. Despite the medicinal benefits of marijuana, its chronic use has been linked with conditions such as psychotic disorders and cannabis use disorder, while acute consumption is linked to psychotic symptoms, hyperemesis syndrome and anxiety (Bridgeman and Abazia 2017).

Therefore research efforts have been intensified to develop several synthetic high-affinity analogs of CB_1_ cannabinoid receptor antagonists and inverse agonists as therapeutic drugs for the management of drug dependence, metabolic syndrome, and diabetes. Literature is replete with inverse agonists of the CB_1_ cannabinoid receptors that have been developed for the management of drug dependence, metabolic syndrome, type 2 diabetes and dyslipidemia (Brown 2007).

Rimonabant, a first-generation synthetic inverse agonist / selective antagonist of the CB_1_ receptor, was approved in Europe in 2006 for the treatment of anorectic obesity (Bridgeman and Abazia 2017). This drug exerts its effect on the ECS by selectively blocking the CB_1_ receptors; thus, reducing appetite and inducing hypophagia. In a randomized double-blind, rimonabant-placebo controlled trial; rimonabant produced a significant reduction in body weights of subjects from 2.6 to 6.3 kg relative to placebo among the groups taking 20 mg of rimonabant daily. HbA_1C_ in obese patients decreased by 0.5–0.6% compared to metformin or sulphonylurea, and 0.8% reduction compared to 0.3% reduction in placebo group. High-density lipoproptein cholesterol (HDL-C) also increased significantly by 22.3% compared with 13.4% in the placebo group while the level of triglycerides decreased in all trials by 6.8% compared with an increase of 8.3% in the placebo group (*p* < 0.0001). The levels of adiponectin, a protein hormone regulating glucose level and fatty acid breakdown in humans, increased significantly by 23% from the baseline in the 20 mg rimonabant group. It was concluded that rimonabant is effective in controlling blood glucose levels and reducing weight in obese patients; however, it was withdrawn from the global market in 2008 due to increased incidences of nausea, upper respiratory tract infections, and serious psychiatric side effects including depression and suicide ideation (Buggy et al. 2011; Christopoulou and Kiortsis 2011; Le Foll et al. 2009). This left a huge research gap as many pharmaceutical companies abandoned the development of inverse CB_1_ receptor agonists. It was opined that the development of novel compounds that are neutral antagonists of the CB_1_ receptor with selectivity for peripheral receptors may be of great value in obtaining similar metabolic results with little or no psychiatric adverse effects. Therefore, research in this area is continuous.

THCV is an inverse agonist / selective antagonist of the CB_1_ receptor, similar to rimonabant but it does not have the identified adverse effects of rimonabant. This short review discusses the potential therapeutic benefits of THCV, a naturally occurring analog of THC, in the management of obesity and type 2 diabetes, its potential side effects, and the mechanism of action within the ECS.

## Methodology

A narrative electronic literature search was performed using peer-reviewed articles published from January 1, 1970, until September 30, 2019. An article was selected if it included keywords such as Δ9-tetrahydrocannabivarin (THCV), Δ9-tetrahydrocannabinol (THC), *Cannabis sativa* (marijuana), obesity, body weight, metabolism, and diabetes. Articles were then reviewed and included based on the applicability to the topic.

## Understanding THCV

THCV is a naturally occurring analog of THC. Unlike THC, which is psychoactive and an agonist at the CB_1_ and CB_2_ receptors, THCV is a non-psychoactive, neutral CB_1_ antagonist / reverse agonist and may act as agonist or antagonist at the CB_2_ receptors depending on its dose. It is thought that THCV prevents the psychological effects of THC however; the mechanism by which THCV antagonizes the effect of THC is unknown. Also unlike THC, THCV produces hypophagic effects in both fasted and non-fasted mice (Riedel et al. 2009). It follows that THCV has great potential for the management of obesity.

The effect of THCV in diet-induced obesity (DIO) and genetic obesity (GO) was evaluated in mice (4 mice per group) using two orally administered dose ranges of THCV stock solution. The solution was appropriately diluted to the required strength using sesame seed oil, for the DIO group at 0.3–12.5 mg/kg twice daily for 30 days and 0.1–12.5 mg/kg once daily for 45 days. One pilot study of 0.3–3 mg/kg per oral once daily; and one full dose range of 0.1–12.5 mg/kg once daily for 30 days in obese mice (Wargent et al. 2013) were also conducted. The results were compared to a potent CB_1_ inverse agonist (AM251) administered per oral at 10 mg/kg once daily or 5 mg/kg twice daily as a positive control. Both doses of AM251 reduced mice’s body weight significantly by greater than 8 g (*p* < 0.001) whereas, THCV did not have any significant effect on the body weight at any of the doses used in the study. Similarly, AM251 decreased the total food intake over the first 10 days of the study, but THCV had no significant effect on the mice’s food intake throughout the study. Neither AM251 nor THCV affected water intake. However, there was a significant reduction in the fat contents by both AM251 (26.4%) and THCV (31.1%) compared to the control (42.1%). There was generally no statistically significant effect on these parameters in the genetically obese mice. It was concluded that similar to AM251, THCV has a high affinity for CB_1_ receptors and high brain penetration, producing some metabolically beneficial effects typical of CB_1_ receptor inverse agonist in two different mouse models of obesity. The strongest effect was on plasma glucose and insulin levels, as well as liver triglycerides. It was opined that THCV may be useful for the treatment of metabolic syndrome and/or type 2 diabetes, either alone or as an adjuvant treatment with other therapeutic options.

Since ECS modulates appetite, food consumption and feeding behavior in animals and humans (Solinas et al. 2008) the acute use of THC, a partial agonist of the CB_1_ receptors, is classically associated with acute appetite-enhancing effects, as well as an increase in the frequency of sucrose ingestion (Jarrett et al. 2005). When THC was administered to rats before the intraoral infusion of sucrose solution, it was noted that THC increased the frequency of sucrose ingestion at 30 and 60 min and particularly, increased palatability at the 120-min interval (Jarrett et al. 2005). Conversely, rimonabant, a CB_1_ antagonist that is similar to THCV, resulted in the reversal of the enhanced frequency of sucrose ingestion and increased palatability (Jarrett et al. 2005).

In a similar report, THCV, a neutral antagonist of the CB_1_ receptors resulted in decreased food intake and body weight reduction in mice models; thus, exerting an anti-obesity effect in mouse models by food aversion (Wargent et al. 2013; Tudge et al. 2015). The metabolic effect of THCV can be explained by its interaction with the transient receptor potential cation channel subfamily V member 1 (TRPV1), also known as the capsaicin receptor (Riedel et al. 2009). Unlike THC, THCV is observed to induce a therapeutic metabolic effect by restoring insulin sensitivity in obese mice models and interacting with the TRPV1 channels (De Petrocellis et al. 2011). THCV has been shown to restore insulin sensitivity in diet-induced obese mice models and reducing obesity by modulating the metabolic processes.

**Fig. 1:**
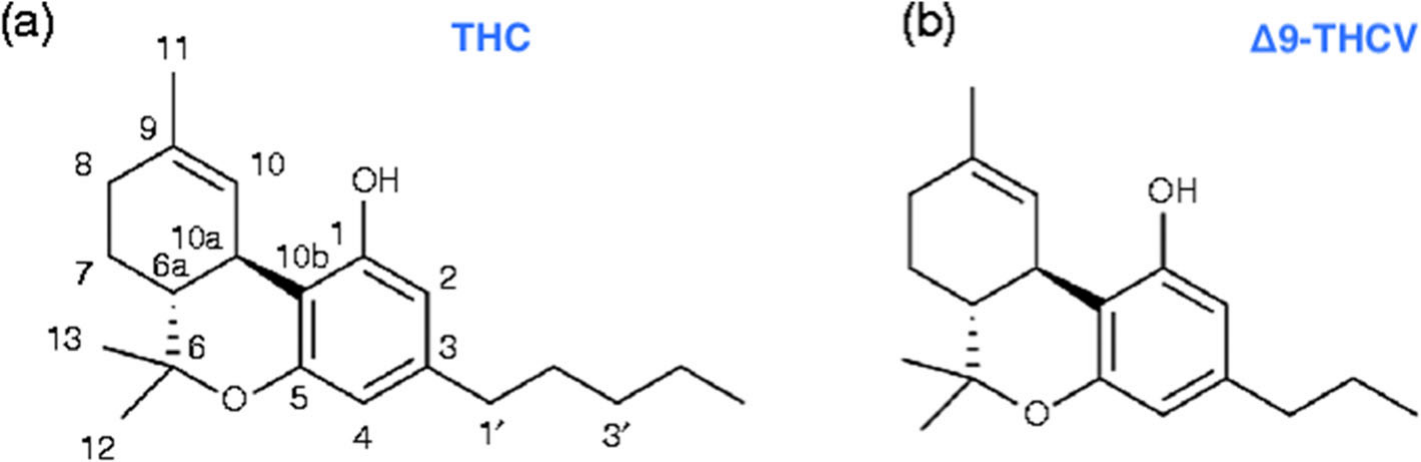
Molecular Structures of THC (**a**), and THCV (**b**). Data sourced from Jager and colleagues in *The Endocannabinoid System and Appetite: Relevance for Food Reward*^19^

The chemical structures of two of the most abundant phytocannabinoids in *Cannabis sativa L*. are highlighted in Fig. 1: THC (a), THCV (b). These phytocannabinoids share some similar structural features that include a dibenzopyran ring and a hydrophobic alkyl chain, but each interacts with the ECS in a slightly different manner (Gill et al. 1970; Jager and Witkamp 2014). Existing in continuous dynamic equilibrium with each other, endocannabinoids are a part of a class of structurally related amides, esters, and ethers of fatty acids (Gill et al. 1970). Although each of these compounds has a slightly different molecular structure, biosynthesis, and physicochemical properties, they all interact with the ECS to maintain homeostasis and regulate lipid and glucose metabolism (Wargent et al. 2013; Jarrett et al. 2005).

For instance, THC and CBD are biosynthesized as tetrahydrocannabinolic acid (THC-A) and cannabidiolic acid (CBD-A) respectively from a common precursor cannabigerolic acid (CBG). These phytocannabinoids are inactive in their natural acidic states but are converted to their respective therapeutically active forms by decarboxylation process when heated. Although they are from the same precursor, THC acts as an agonist at the cannabinoid receptors and results in an increased lipid and glucose intake (McPartland et al. 2015; Jarrett et al. 2005; Jager and Witkamp 2014), whereas THCV exhibits antagonistic activities at the cannabinoid receptors (Thomas et al. 2005). Studies using mice models have indicated dose-dependent therapeutic effects (Jadoon et al. 2016). At low intravenous doses (0.1, 0.3, 1.0 and/or 3 mg/kg), the plant-derived THCV and its synthetic analogs (O-4394 and O-4395) show antagonism at the cannabinoid receptors by reversing some of the effects of THC, such as THC-induced antinociception and hypothermia (Pertwee et al. 2007). THC activates both peripheral and central CB_1_ receptors (Muniyappa et al. 2013) when administered alone. At higher doses, both O-4394 and O-4395 exhibit agonistic effects at the cannabinoid receptors by precipitating hypothermia (above 3 mg/kg) and antinociception (above 10 mg/kg) (Pertwee et al. 2007). The cannabinoid receptors and their ligands have been implicated in feeding and metabolic control regulations (Cluny et al. 2015; Ravinet-Trillou et al. 2004) providing a potential therapeutic benefit for the treatment of type 2 diabetes in the human population.

A significant increase in body weight (24%) and adiposity (60%) in CB_1_ +/+ mice compared to the CB_1_ −/− mice has been reported when both groups were fed with standard diet containing 3.5 kcal/g and 14.5% of energy as fat (Ravinet-Trillou et al. 2004). However, when both types of mice were fed with a high-fat obesity-prone diet containing 4.9 kcal/g and 49% of energy as fat, CB_1_ −/− mice did not develop obesity in contrast to the CB_1_ +/+ mice in spite of the similar energy intake. This suggests an improved metabolic regulation in the CB_1_ −/− mice (Ravinet-Trillou et al. 2004). In another study, fasting plasma glucose levels and oral glucose tolerance test (OGTT) improved in mice with diet-induced obesity when plant-derived THCV was administered twice daily (Wargent et al. 2013). Administration of intraperitoneal plant-derived THCV in rodents resulted in weight loss, reduced food intake, reduced body fat content, increased energy expenditure, rapid insulin response to OGTT (Wargent et al. 2013), and reduced liver triglycerides (Ravinet-Trillou et al. 2004; Englund et al. 2015).

Similar to the rimonabant human clinical trials mentioned above, the selective CB_1_ receptor antagonist rimonabant, exhibited potent anti-obesity properties in CB_1_ (+/+) obese mice leading to leanness and hypophagia (Wargent et al. 2013; Ravinet-Trillou et al. 2004). In Zucker rats, rimonabant reduced the levels of plasma triglycerides, free fatty acids, total cholesterol, and increased the levels of high-density lipoprotein/low-density lipoprotein (HDL/LDL) ratio (Thomas et al. 2005). Similar effects on lipid profiles were observed when a high dose of the plant-derived THCV (12.5 mg/kg) was administered to diet-induced obese mice once daily (Wargent et al. 2013). There was no significant change in the glycemic profile until after 3 weeks of administering high dose plant-derived THCV (12.5 mg/kg), where the once-daily administration of THCV resulted in a lower fasting glucose and the twice-daily administration of THCV resulted in increased glucose intolerance (Wargent et al. 2013). This suggests that THCV has a more profound leptin-based effect on the lipid profile than the glucose profile in both fasting and non-fasting states. In CB_1_ knockout mice, rimonabant does not display the anti-obesity properties that were previously observed in diet-induced obese mice (Ravinet-Trillou et al. 2004). Like THCV, other synthetic cannabinoid antagonists such as O-4394 and O-4395 (Ravinet-Trillou et al. 2004; Englund et al. 2015), modulate the cannabinoid receptor activity. They showed similar physiologic activity, displacing the (3)-H-CP55940 in the mouse brain and antagonizing specific activity at the CB_1_ receptor sites in the brains of mice and vas deferens (CP55940 and R-(+)-WIN55212), respectively (Anavi-Goffer et al. 2012).

In a placebo-controlled, double-blind, cross-over pilot study involving ten male cannabis users (less than 25 uses/occasion), 10 mg pure THCV or placebo was given for 5 days followed by 1 mg intravenous THC infusion on the last day. When a low dose of oral THCV was administered before the THC intravenous dose, THCV blunted the well-known effects of THC including psychotic and paranoia effects, and impaired short-term memory (Englund et al. 2015).

In another randomized, double-blind, placebo-controlled, parallel-group pilot study, the safety and efficacy of THCV and CBD were evaluated in patients with type 2 diabetes using the glycemic and lipid parameters. Sixty-two patient volunteers with non-insulin treated type 2 diabetes were randomized to five treatment groups viz.: CBD (100 mg twice daily), THCV (5 mg twice daily), 1:1 ratio of CBD and THCV (5 mg/5 mg, twice daily), 20:1 ratio of CBD and THCV (100 mg/5 mg, twice daily) and matched placebo for 13 weeks. Patients were at least 18 years of age with hemoglobin A1C (HbA_1C_) levels less than 10% (Jadoon et al. 2016).

THCV significantly decreased fasting plasma glucose (from 7.4 to 6.7 mmol/L) compared to the placebo group which increased from 7.6 to 8 mmol/L ^21^ with an estimated treatment difference (ETD) of − 1.2 mmol//L, *p* < 0.05. It also improved the Homeostasis Model Assessment (HOMA2) of pancreatic β-cell function from 105.1 to 144.4 points compared to 96.4 to 94.7 points in the placebo group (ETD = 44.6 ± 16.1, *p* < 0.01) (Jadoon et al. 2016). Adiponectin is the protein hormone involved in regulating the plasma glucose levels and fatty acid breakdown (pancreatic function). The pancreatic β-cell function improved significantly in the THCV treatment group relative to placebo (ETD = − 5.9 × 10^6^ pg/mL, *p* < 0.01), as well as apolipoprotein A (ETD = − 6.02 μmol/L, *p* < 0.05), but there was no significant effect on the HDL cholesterol. CBD decreased resistin significantly (− 898 pg/mL, *p* < 0.05) and increased glucose-dependent insulinotropic peptide (21.9 mL, p < 0.05) compared to the baseline.

It was concluded that THCV and CBD alone and their combination products were well-tolerated in patient volunteers with type 2 diabetes. THCV significantly decreased the fasting plasma glucose, increased β-cell function, as well as adiponectin and Apo A concentrations in type 2 diabetic patients. It was evident that THCV may provide a template for the development of new therapeutic agents for glycemic control, especially for type 2 diabetics.

From the foregoing, it is obvious that the non-psychoactive effect of THCV provides a therapeutic advantage over other cannabinoid analogs in addition to its hypoglycemic and hypolipidemic effects. Hence, further intensive research is urgently needed to produce clinically useful medicinal agents from THCV derived from marijuana (*Cannabis sativa)*. As shown from this short review, it is important to emphasize that the pure plant-derived THCV did not elicit the common adverse effects associated with rimonabant (psychiatric and anxiogenic-like reaction) and AM251 (nausea) (McPartland et al. 2015) reported in this review. Although the reason for this difference is not fully understood it was hypothesized that THCV might competitively inhibit one of the signaling pathways of one or more endogenously produced endocannabinoids through CB_1_ receptor activity (McPartland et al. 2015). Another explanation for the anti-obesity feature of THCV can be attributed to its ability to interact with other receptor sites, including the G-protein-coupled receptor (GPR55)^27,^ the transient receptor potential vanilloid 1 receptor (TRPV1) (De Petrocellis et al. 2011) and other endogenous endocannabinoids for the receptor site (Riedel et al. 2009). A summary of the effects of THCV on human and mouse/animal: metabolism, glycemic and lipidemic responses are highlighted in Table 1.

**Table 1:** Summarized Metabolic, Glycemic, and Lipidemic Effects of THCV

	Metabolic	Glycemic	Lipidemic
THCV Effects			
Human Studies	Increase FFA suppression index (FFA auc/Insulin auc) (Muniyappa et al. 2013)	Induces glucose intolerance in men (Muniyappa et al. 2013) Impaired adipose tissue insulin sensitivity (Muniyappa et al. 2013) Increase indices of adipose tissue insulin resistance (Muniyappa et al. 2013) Normal glucose tolerance due to no impairments on β-cell glucose sensitivity, rate sensitivity, or insulin secretion (Muniyappa et al. 2013) Decreased fasting plasma glucose (Jadoon et al. 2016) Improved pancreatic β-cell function (Jadoon et al. 2016)	No difference in total cholesterol level (Muniyappa et al. 2013) Lower plasma HDL level (Muniyappa et al. 2013) vs. plasma HDL unaffected (Jadoon et al. 2016) No difference in LDL cholesterol (Muniyappa et al. 2013) No difference in triglycerides (Muniyappa et al. 2013) No difference FFA levels (Muniyappa et al. 2013)
Animal Studies	Improved fasting plasma glucose (Wargent et al. 2013)	Pancreatic CB1R activation leads to β-cell death and impairs insulin secretion (Muniyappa et al. 2013) Improved glucose tolerance (Wargent et al. 2013) Increased insulin sensitivity (Wargent et al. 2013) Restores insulin sensitivity in cells that are insulin-resistant (Wargent et al. 2013)	Increase adipocyte hypertrophy - increase hepatic fat (Muniyappa et al. 2013) Increase in lipogenesis (Muniyappa et al. 2013) No effect on plasma total cholesterol and triglyceride (Wargent et al. 2013) No change in HDL cholesterol concentrations (Wargent et al. 2013)

Note: Data sourced from Muniyappa (Muniyappa et al. 2013) and colleagues, Wargent (Wargent et al. 2013) and colleagues, and Jadoon (Jadoon et al. 2016) and colleagues

## Conclusion

The psychoactive effects of THC in marijuana are the main reasons for its classification as a Schedule I substance, even though it is the THC that the U.S. Food and Drug Administration (FDA) approved for appetite stimulation and weight gain. In contrast to THC, clinical and therapeutic advantages of THCV regarding its lack of psychoactive effects in human studies are of great value in pharmacotherapy. On the other hand, the dual pharmacological activities of THCV on CB_1_/CB_2_ receptors, exhibiting agonistic and antagonistic effects depending on the dosage, indicate the need for further research. It is envisioned that the unique and diverse characteristics of THCV could be explored for further development into clinically useful medicines for the treatment of life-threatening diseases.

## Data Availability

Not applicable

## Abbreviations

ASD: Autism Spectrum Disorder
CB: Cannabinoid receptors
CBD: Cannabidiol
CB1,2: Cannabinoid type 1,2 receptors
CBD-A: Cannabidiolic acid
CBG: Cannabigerolic acid
DIO: Diet induced obesity
ECs: Endocannabinoid system
ETD: Estimated Treatment Difference
FDA: Food and Drug Administration
GPR55: G protein coupled receptor
GO: Genetic obesity
HbA1C: Hemoglobin A1C
HDL: High density lipoprotein
HDL-C: High density lipoprotein cholesterol
HOMA2: Homeostasis Model Assessment
LDL: Low density lipoprotein
OGTT: Oral glucose tolerance test
THC: Tetrahydrocannabinol
THCV: Δ9 Tetrahydrocannabivarin
TRPV1: Transient receptor potential vanilloid 1 receptor
THC-A: Tetrahydrocannabinolic acid
